# Effect of zinc imprinting and replacing inorganic zinc with organic zinc on early performance of broiler chicks

**DOI:** 10.3382/ps/pew312

**Published:** 2016-09-23

**Authors:** S. Mwangi, J. Timmons, T. Ao, M. Paul, L. Macalintal, A. Pescatore, A. Cantor, M. Ford, K. A. Dawson

**Affiliations:** * Food Science and Technology Program, Department of Agriculture, Food and Resource Sciences University of Maryland Eastern Shore, Princess Anne, Maryland 21853; † Alltech-University of Kentucky Nutrition Research Alliance, Lexington, Kentucky 40546

**Keywords:** imprinting, zinc, broiler, organic, inorganic

## Abstract

The goal of this study was to determine the effects of feeding a zinc (Zn) deficient diet to broiler chicks for 96 h post-hatch followed by feeding diets with different Zn sources and supplemental levels (5 to 21 d) on the growth performance, tissue, and excreta Zn content. At the start of the study, four hundred 20-day-old male broiler chicks were divided into two groups. One group was fed a corn soybean meal based diet containing 25 mg of Zn/kg (imprinting diet, ID). The second group was fed the basal diet supplemented with 40 mg of Zn/kg from Zn oxide (ZnO) (non-imprinting diet, NID). Both groups were fed these diets for 96 h. At d 5, chicks from each group were randomly assigned to the dietary treatments consisting of the basal diet alone or the basal diet supplemented with 8 or 40 mg/kg Zn as ZnO or Zn proteinate. Main effects of post-hatch Zn ID were observed on feed intake and G:F. ID decreased (*P* < 0.05) feed intake and improved (*P* < 0.05) the gain to feed ratio (G:F) of 14 and 21 d old chicks compared to G:F of chicks fed NID. Additionally, G:F for 14 and 21 d was improved (*P* < 0.05) by interaction of Zn source × level. Furthermore, at d 21 chicks fed the ID had a lower (*P* < 0.05) Zn content in the tibia ash and excreta, and a higher (*P* < 0.05) Zn content in the pancreas tissue compared to chicks fed NID. These results suggest that Zn imprinting can affect body Zn stores and early performance.

## INTRODUCTION

Zinc (Zn) is an important trace mineral required in animal nutrition for different biological activities (Suttle, 2010). The recommended Zn requirement that is based on performance criterion for broiler chickens is 40 mg/kg of diet Nation Research Council (NRC, 1994). However, NRC recommended values for most trace minerals are based on older strains of broilers and may be outdated for the modern strains of broilers used in commercial production today (Leeson, 2005). Traditionally, inorganic trace minerals such as oxides and sulfates are supplemented in broiler diets above the NRC recommended level to maximize performance (Leeson, 2005; Leeson and Caston, 2008). When inorganic trace minerals are fed and reach the upper gastrointestinal tract they tend to dissociate due to the low pH environment. These dissociated minerals can interact with other minerals as well as other dietary components in the digesta, which make them unavailable for absorption across the small intestine (Wedekind and Baker, 1990; Underwood and Suttle 1999; Leeson and Summers 2001; Yan and Waldroup, 2006). As a result, these unabsorbed and excess minerals not utilized by the birds are excreted in the feces, and may lead to environmental concerns when poultry manure is applied to cropland as fertilizer (Gupta and Charles, 1999; Leeson 2003; Aksu et al., 2010; Kibet et al., 2013)

Dietary strategies should be implemented to avoid overfeeding dietary nutrients without jeopardizing animal health and performance (Spears, 1996; Ferket et al., 2002). One dietary strategy that may be utilized to reduce over supplementation of trace minerals is replacing inorganic trace minerals with organic sources. Results from different studies have indicated that the use of organic trace minerals in broiler diets can enhance mineral uptake, improve body weight gain, and reduce mineral excretion (Burrell et al., 2004; Ao et al., 2006; Yan and Waldroup, 2006; Bao et al., 2007; and Nollet et al., 2007). The high bioavailability of organic trace minerals can be explained in part by their stability in the upper gastrointestinal tract, which allows the minerals to reach the small intestine where they are absorbed (Ashmead 1993). However, not all organic minerals are stable at a low pH, which may affect their bioavailability (Cao et al., 2000; Guo et al., 2001).

In addition to using organic trace minerals, recent studies have indicated that early life dietary manipulation can influence the utilization of the restricted nutrient later in life. For instance, broiler chickens subjected to early feed restriction from 1 to 21 d for four hours per day had poor growth performance and lipid metabolism. Although, the birds were provided ad libitum access to feed from 22 to 63 d, the feed restricted broilers were more fatty and obese compared to broilers that had free access to feed and water (Zhan et al., 2007). The intestinal gene expression profile of broiler chicks fed a diet supplemented with 100% NRC levels of organic trace minerals (Zn,Cu, and Mn), from 1 to 4 d and later (5 to 21 d) fed a diet supplemented with 20% of the NRC (1994) recommended level, was different from that of broiler chicks fed a diet supplemented with 20% NRC level of organic trace minerals from 1 to 21 d (Brennan et al., 2013). Yan et al. (2005) reported that broiler chicks fed diet low in phosphorus (P) and calcium (Ca) from hatch to 18 d of age, absorbed more P and Ca later in life (18 to 32 d) than birds fed the recommended P and Ca levels. Post-hatch dietary manipulation and replacement of inorganic trace minerals with low levels of organic trace minerals have shown to affect broiler performance and development. However, it is unknown if feeding a Zn deficient diet to broiler chicks 96 h post hatch has a preconditioning effect on Zn absorption in broiler chicks.

Therefore, the objective of this study was to determine the effect of Zn imprinting and supplementing different forms and levels of Zn in broiler diets on growth performance, tissue mineral concentration, and Zn excretion.

## MATERIAL AND METHODS

### Chicks and Housing

Four hundred and twenty 1-day-old (Cobb 500) male broiler chicks were used in this study. Six replicates of seven chicks per treatment were randomly allotted by weight to mesh wire floored cages (61 cm × 51 cm × 36 cm) containing two adjustable nipple drinkers and one plastic feeder. Temperature was maintained at 31°C for the first week and then decreased and maintained at 27°C to the end of the trial. Chicks were provided 24 hours of light throughout the trial, and allowed free access to mash feed and water. The water was analyzed, and contained Zn below detectable levels. All experimental procedures complied with the University of Kentucky Institutional Animal Care and Use guidelines.

### Experimental Design and Treatments

The experimental design was a randomized complete block in which each cage was blocked based on room location, and considered an experimental unit. The basal corn-soybean meal diet (Table 1) was formulated to meet or exceed the NRC requirement (NRC, 1994) for all nutrients except for Zn. Organic Zn was added to the diets as Bioplex Zn, (Alltech Inc. Nicholasville, KY) and inorganic Zn was added as technical grade ZnO. The diet was not supplemented with phytase. During the imprinting period (1 to 4 d), chicks were assigned to either the basal diet analyzed to contain 25 mg/kg of Zn (imprinting diet, ID) or basal diet supplemented with 40 mg Zn/kg of diet as ZnO (non-imprinting diet, NID). At 5 d of age, six replicates cages (6 chicks/cage) from each group were randomly assigned to the dietary treatments consisting of the basal diet alone or the basal diet supplemented with 8 or 40 mg/kg Zn as ZnO or Bioplex Zn (10 treatments with 6 replicates). The formulated and analyzed Zn concentrations in the diets are presented in Table 2.

**Table 1.** Composition of the basal diet.

*Ingredient:*	%
Corn	59.36
SBM, dehulled (48% CP)	33.70
Corn Oil	3.00
Dicalcium phosphate (Technical grade)	1.47
Calcium carbonate (Technical grade)	1.21
Salt, iodized	0.45
DL-methionine	0.21
L-lysine	0.10
Vitamin premix^1^ (no mineral)	0.25
Mineral premix^2^ (no Zn)	0.25
Total	100.00
Calculated (analyzed) nutrients	
ME, kcal/kg	3,080
CP, %	21
Ca^3^, %	1.00 (1.3)
Nonphytate P^3^, %	0.45 (0.40)
Lysine, %	1.24
Methionine + Cystein, %	0.90
Zinc (mg/kg)	25

^1^Vitamin premix per kilogram of diet: 11,025 I.U. vitamin A, 3,528 I.U. vitamin D3, 33 I.U. vitamin E, 0.91 mg vitamin K, 2 mg thiamin, 8 mg riboflavin, 55 mg niacin, 18 mg Ca pantothenate, 5 mg vitamin B.

^2^Mineral premix per kilogram of diet: 0.264 mg Se as Na_2_SeO_3_; 12.80 mg Cu as CuSO_4_.5H_2_O; 0.24 mg I as KIO_3_; 106.67 mg Fe as FeSO_4_-H_2_O;81.36 mg Mn as MnSO_4_-H_2_O.

^3^Determined by analysis of duplicate samples.

**Table 2.** Calculated and analyzed Zn content of the broiler diets.

Zn	Supplemented	Analyzed Zn
source	Zn (mg/kg)	content (mg/kg)^a^
Basal diet	0	25.00
ZnO	8	39.18
Bioplex Zn	8	32.64
ZnO	40	68.46
Bioplex Zn	40	70.29

^a^Analyzed Zn values are based on chemical analysis of triplicate samples of each diet.

### Broiler Performance

Body weight gain (**BWG**) and a record of the total amount of feed consumed (**FC**) per cage were recorded on day 1, 5, 14, and 21. Feed was weighed and non-consumed feed was weighed back at the end of each period except at 5 d. Gain to feed ratio (**G:F**) at 5, 14, and 21 d of age was determined by dividing the total bird weight adjusted for mortality per cage by the amount of feed consumed.

### Tissue Sampling and Preparation

At d 5 and 21, liver samples were collected from one bird of average weight from each cage (six birds per treatment) for Zn concentration analysis. At d 21, right tibia bones from two birds/cage were pooled and boiled in deionized water for approximately 10 min to remove of all soft tissues. The bones were then dried at 60°C for 72 h, and fat extracted in petroleum ether for 3 d. The defatted tibia bones were dried for 12 h at 105°C, and cooled to room temperature in desiccators. The dried tibias were placed in a muffle furnace at 600°C overnight for ash determination. Pancreas tissues were collected from six birds per treatment for Zn concentration analysis.

Excreta samples were collected on d 21 for 24 h from each replicate using collection trays. Non-excreta material was removed, and the excreta from each cage was pooled, weighed, and dried for 48 h at 105°C. Before mineral analysis, the dried excreta and feed samples were ground into fine particles using a coffee grinder and sealed in plastic bags. Samples were stored at 4°C until they were analyzed for Zn content.

### Acid Digestion and Mineral Determination

Before mineral analysis, approximately 1 g of the freeze-dried, homogenized liver and pancreases samples as well as tibia ash, excreta, and feed samples were microwave digested with nitric acid for analysis of Zn using inductively coupled plasma emission spectroscopy (ICP-OES, axial 720 series) according to method described in AOAC, (1996).

### Statistical Analysis

To determine the effect of supplemental Zn, a single degree of freedom contrast was used to compare all supplemental levels with control (Li et al., 2011). Data for all variables excluding the control were further analyzed by three way analysis of variance (ANOVA) with the model including the main effect of Zn level, source, imprinting and their interaction, using general linear model of Statistix V. 9 (Analytical Software, Tallahassee, FL). Tukeys-HSD was used to determine means differences among treatments. Significant differences among treatments were determined at probability of *P* ≤ 0.05.

## RESULTS

### Performance

The Zn imprinting diet did not affect (*P >* 0.05) BWG, FC, and G:F from 0 to 5 d when compared to birds fed the non-imprinting diet (63.9 g/bird, 97.4 g/bird and 0.658 vs. 63.4 g/bird, 98.9 g/bird and 0.641, respectively). At 14 and 21 d of age, there was a Zn level by source interaction on the G:F. Broiler chicks fed a diet supplemented with 40 mg/kg of ZnO had significantly improved G:F compared to those fed a diet supplemented with 8 mg/kg of ZnO and 40 mg/kg of Bioplex Zn, but not those fed diet supplemented with 8 mg/kg of Bioplex Zn. Early Zn imprinting diet increased (*P* < 0.05) the G:F and lowered (*P* < 0.05) FC at 14 and 21 d when compared with the non-imprinted diet (Table 3).

**Table 3.** Effects of Zn imprinting^1^ diet for the first 96 h post hatch and replacing zinc oxide with Bioplex Zn on growth performance of broiler chicks.

	Weight gain g/bird		Feed intake g/bird		Feed efficiency G:F	

Item	1 to 14 d	1 to 21 d	1 to 14 d	1 to 21 d	1 to 14 d	1 to 21 d
Imprinting^2^						
Zn imprinted	404	812	555^b^	1202^b^	0.73^a^	0.68^a^
Non-imprinted	403	802	571^a^	1223^a^	0.70^b^	0.65^b^
Pooled SE	2.55	5.07	4.10	7.31	0.01	0.01
Zinc source						
Basal diet^3^	408	810	561	1204	0.73	0.67
Zinc Oxide^2^	404	809	563	1214	0.72	0.67
Bioplex Zn	401	803	564	1216	0.71	0.66
Pooled SE	2.55	5.07	4.10	7.31	0.01	0.01
Zn Level (mg/kg)^2^						
8	401	802	567	1217	0.71	0.66
40	404	810	559	1211	0.72	0.67
Pooled SE	2.2	5.07	4.4	7.31	0.01	0.01
Interaction of Zn level × Zn source						
8 mg/kg Zinc Oxide	401	797	574	1221	0.70^b^	0.65^b^
8 mg/kg Bioplex Zn	402	807	562	1214	0.72^a,b^	0.67^a,b^
40 mg/kg Zinc Oxide	406	821	552	1206	0.74^a^	0.68^a^
40 mg/kg Bioplex Zn	401	799	567	1217	0.71^b^	0.66^b^
*P*-values						
Imprinting	0.950	0.210	0.050	0.050	0.014	0.050
Zn source	0.618	0.563	0.879	0.866	0.557	0.504
Zn level	0.683	0.417	0.246	0.633	0.153	0.229
Imprinting source	0.564	0.752	0.780	0.074	0.826	0.267
Imprinting × level	0.313	0.591	0.656	0.755	0.717	0.449
Source × level	0.524	0.138	0.067	0.462	0.025	0.034
Imprinting × source × level	0.509	0.612	0.160	0.884	0.413	0.470

^1^No supplemental Zn in the basal diet fed to chicks for 96 h.

^2^Data represent means of 24 replicates cages.

^3^Data present means of 12 replicates cages.

^a,b^Means within the same column within the same factor with any identical letters are not significantly different at *P* ≤ 0.05 by Tukeys-HSD.

### Tissue Mineral Concentration

Broiler chicks fed the imprinting diet (basal diet) had a lower (*P <* 0.05) liver Zn content on 5 d compared to those fed the non-imprinting diet (42.75 vs. 50.14 mg/kg, respectively). Pancreatic Zn concentrations in broiler chicks fed the imprinting diet was higher (*P* < 0.05) compared to the birds fed the non-imprinting diet. Chicks fed diets with Bioplex Zn supplementation had a higher (*P* < 0.05) pancreas Zn content compared to the pancreas Zn content of birds fed the basal diet alone or basal diet supplemented with ZnO. Chicks fed the basal diet supplemented with 40 mg/kg of Zn had higher (*P* < 0.05) pancreatic Zn concentration compared to birds fed the basal diet or supplemented with 8 mg/kg of Zn. (Table 4).

**Table 4.** Effects of zinc imprinting^1^ and replacing zinc oxide with Bioplex Zn at different levels on tissues and excreta Zn from broiler chicks raised to 21 d.

Item	Tibia ash Zn (mg/kg)	Tibia ash (%)	Excreta Zn (mg/kg)	Liver Zn (mg/kg)	Pancreas Zn (mg/kg)
Imprinting^2^					
Zn imprinted	420.91^b^	52.47	175.02^b^	67.25	84.67^a^
Non-imprinted	460.23^a^	53.34	180.79^a^	65.63	74.67^b^
Pooled SEM	11.56	0.46	8.28	1.01	2.98
Zinc source					
Basal diet^3^	335.26^b^	52.60	98.88^b^	65.16	61.46^c^
Zinc oxide^2^	420.76^a^	53.18	175.09^a^	64.92	73.8^b^
Bioplex Zn^2^	460.37^a^	52.76	178.14^a^	68.04	85.54^a^
Pooled SEM	11.56	0.46	8.28	1.01	2.98
Zn level (mg/kg)^2^					
8	402.70^b^	52.75	122.05^b^	65.52	67.10^b^
40	478.44^a^	53.18	233.76^a^	67.45	92.15^a^
Pooled SEM	11.56	0.46	8.27	1.01	2.90
*P*-values					
Imprinting	0.028	0.205	0.047	0.400	0.019
Zn source	0.001	0.655	0.05	0.092	0.001
Zn level	0.001	0.678	0.0001	0.292	0.001
Imprinting × source	0.321	0.519	0.751	0.143	0.763
Imprinting × level	0.979	0.491	0.634	0.238	0.101
Source × level	0.090	0.532	0.428	0.080	0.377
Imprinting × source × level	0.819	0.114	0.286	0.306	0.290

^1^No supplemental Zn in the basal diet fed to broiler chicks for 96 hours.

^2^Data represent the means of 24 replicates cages.

^3^Data represent the means of 12 replicates cages.

^a–c^Means with different superscript in the same column are significantly different at *P* ≤ 0.05 by Tukeys-HSD.

### Excreta Zn, Tibia ash Zn, and Total Tibia Ash

At 21 d, the Zn excreta content from broiler chicks fed the Zn imprinting diet was significantly lower (*P* < 0.05) compared to that of broiler chicks fed the non-imprinting diet. Broiler chicks fed the basal diet supplemented with 40 mg/kg Zn had a significantly higher (*P* < 0.05) Zn excreta content compared to the Zn excreta content from chicks fed a diet supplemented with 8 mg/kg of Zn. The Zn sources had no effect (*P* > 0.05) on excreta Zn content; however, excreta Zn content from the two sources was significantly different from the basal diet (Table 4).

Broiler chicks fed the imprinting diet had significantly lower (*P* < 0.05) tibia ash Zn concentration than those fed the non-imprinted diet (Table 4). The tibia ash Zn concentrations from broiler chicks fed diet supplemented with 40 mg/kg of Zn was higher (*P* < 0.05) than that of chicks fed the diet supplemented 8 mg/kg of Zn. There were no significant differences in the tibia ash Zn content and percentage tibia ash from birds fed the inorganic and organic source of supplemental Zn.

## DISCUSSION

### Performance

Dietary manipulation early in life has been shown to affect broiler performance (Sklan and Noy 2000; Zhan et al., 2007). In the present study, the imprinting diet fed to broiler chicks (1 to 4 d) did not affect performance at 5 d. These results could be explained in part by the fact that internalized egg yolk into the body cavity of broiler chicks towards the end of incubation period, provide immediate nutrition required for growth post-hatch (Richards 1997; Oliveira et al., 2015). Additionally, these results may suggest that the Zn levels in the imprinting diet were not low enough to induce immediate effect on performance. However, our data showed that the Zn imprinted group at 14 and 21 d had significantly improved feed efficiency and a lowered (*P* < 0.05) FC and numerically improved body weight at 21 d of age compared to that of the chicks in the non-imprinted group. These results could be attributed to efficient use of supplemental Zn by broiler chicks after the Zn imprinting period, which was demonstrated by increased pancreas Zn concentrations, and reduced excretion of Zn in the feces. However, while Zn supplementation to a broiler diet has been shown to improve broiler performance, it should be noted other factors could influence performance. Hence, performance alone may not be a good index of the Zn requirement for broiler chicks especially when a corn soybean meal diet is fed to broiler chicks, this is likely due to the fact that this ingredients may contain minimum Zn level required for growth. (Wedekind et al., 1992; Huang et al., 2007; Sunder et al., 2011).

In the present study, added Zn levels and the source of Zn had no significant effect on FC and BWG however; their interaction had significant effect on feed efficiency at 14 and 21 d. The addition of 40 mg/kg of ZnO promoted increased feed efficiency (*P* < 0.05) compared to the supplementation of 8 mg/kg of ZnO and 40 mg/kg of Bioplex Zn. The lack of statistical difference in FC and BWG indicated that the amount of Zn in the basal diet, which was analyzed to be 25 mg/kg, was adequate to support optimal growth during the 21 d post-hatch period despite the NRC (1994) Zn recommendation of 40 mg/kg of Zn/kg for broiler chicks. These results were in agreement with those obtained by Rossi et al. (2007) and Vieira et al. (2013) which indicated that the absence of supplemental Zn to a broiler diet did not affect bird performance. Similarly, Sunder et al. (2008) reported that a basal diet containing 29 ppm Zn was enough to support maximum bird performance. Additionally, as suggested by Star et al. (2012), lack of response on FC and BWG in our study, could be due to the short duration of the experiment and imprinting period, and future research to test the effect of prolonged imprinting periods on performance will be of interest.

### Tibia ash Zn and Percentage Bone ash Content

Huang et al. (2009) and Ao et al. (2009) reported that dietary level and source of the trace minerals significantly affected bone mineral concentration. Studies by Wedekind et al. (1992) and Baker and Ammerman (1995) indicated that bone Zn is the most sensitive index for Zn bioavailability in broiler chickens regardless of the amount of supplemental Zn in the diet. Based on the tibia ash Zn concentration, Ao et al. (2006) indicated that the relative bioavailability of Bioplex Zn was 157% compared to Zn sulfate (100% bioavailable). Cao et al., (2000), using multiple linear regression slope ratio of bone Zn, indicated that the bioavailability of Zn proteinate was 139% when the bioavailability of Zn sulfate was set at 100%. Bao et al. (2007) reported that tibia Zn concentrations were strongly related to the dietary organic Zn intake (R^2^ = 70.28%). Using the tibia ash Zn content as a predictor of bioavailability, the results from the current study indicated that the bioavailability of ZnO and Bioplex Zn were similar to each other, although the value of the Zn tibia ash content from chicks fed the Bioplex Zn supplemented diet was numerically higher than Zn tibia ash content of ZnO).

Bones have been established as functional reserve of Zn in broiler chickens, which can be used during Zn deficiency (Harland et al., 1975; Suttle, 2010). Results from the current study indicate that tibia ash Zn content from birds fed the Zn imprinting diet (basal diet) 1 to 21 d was lower compared to the tibia ash Zn content from the other treatments supplemented with Zn. Similarly, Vieira et al. (2013) reported that the tibia ash Zn concentration was significantly decreased when chicks were fed a diet with 0 ppm supplemental Zn. The present data indicate that feeding broiler chicks a Zn imprinting diet can permanently alter tibia ash Zn content.

In the present study, Zn imprinting, Zn level and source did not affect the tibia ash percentage. These results were in agreement with those obtained by Sahraei et al. (2012), which indicated that the supplementation of 100, 150, and 200 mg/kg of Zn as either ZnO (72% Zn) or Bioplex Zn (15% Zn) to a corn-soybean diet fed to broiler chicks did not have a significant effect on the ash percentage. Similarly, Sunder et al. (2008) also reported that the tibia ash content from chicks did not vary with Zn level in the feed up to 160 ppm. However, there was a significant decrease in the percent tibia ash from chicks fed a diet supplemented with 320 ppm Zn, which was attributed to negative effect of higher Zn levels on bone mineralization. Yi et al. (1996) reported that toe and tibia ash percentage of broiler chicks were not affected by adding Zn to broiler diets.

### Concentrations of Zn in the Liver and Pancreas

Tissue mineral concentrations can be used as indicators of body mineral storages (Wedekind et al., 1992). In the present study, broiler chicks fed the Zn imprinted diet had a lower (*P* < 0.05) level of liver Zn content compared to those fed the non-imprinting diet from 1 to 4 d of age (basal diet supplemented with 40 mg/kg of ZnO). However, the analysis of the 21 d liver Zn concentrations indicated there were no effect (P > 0.05) of Zn imprinting, source, and level. These results are similar to those obtained by Johnson et al. (1988), which indicated that feeding a deficient Zn diet to rats followed by feeding a Zn adequate diet helped the rats overcome their deficiency. Results from our study indicated that the liver Zn content from broiler chicks fed the Zn imprinted diet (1 to 4 d) did catch up to the levels of Zn found in 21 d old broiler chicks fed the non-imprinted diet. This observation is supported by the significantly lower Zn excreta levels from broiler chicks fed the Zn imprinted diet. Bao et al. (2007) reported that concentration of Zn in the liver of birds fed a control diet (basal diet without supplemental Zn) was higher (*P* < 0.05) than that from birds fed diet supplemented with different levels of Zn. Ao et al. (2007) indicated that there was linear response of the liver Zn content due to increased supplemental Zn levels from Bioplex Zn or Zn sulfate in the diet, although the increase was not significantly different between the two sources. Sunder et al. (2008) and Sandoval et al. (1998) also reported a linear increase (*P* < 0.05) in the liver Zn deposition from broiler chicks fed a diet supplemented with graded levels of Zn sulfate. However, Sunder et al. (2013) reported that the Zn and Mn retention in the hepatic tissues from broiler chicks increased significantly (*P* < 0.05) only in the chicks fed the 160 and 240 mg/kg, respectively supplemented diets. These researchers concluded that higher dietary Zn and Mn supplementation were required for a significant increase of mineral concentration in the hepatic tissue.

The pancreas is both an endocrine and exocrine organ, which has a unique Zn requirement for biological processes, which include, production of digestive enzymes as well as insulin packaging, secretion, and signaling. (Shannon et al., 2011). Huang et al. (2007) reported that the pancreas is the most sensitive soft tissue in response to dietary Zn concentration fed to broiler chicks. In the present study, there was a significant main effect (*P* < 0.05) of Zn imprinting, source, and level on the pancreas Zn levels. Broiler chicks fed the Zn imprinted diet had higher (*P* < 0.05) pancreatic Zn content compared to the pancreatic Zn content from chicks fed the non-imprinted diet. This observation could be explained by the fact that during marginal Zn deficiency, there is increased Zn mobilization from the liver back into circulation for maintenance of Zn homeostasis especially for tissues like pancreas, which have unique Zn requirement (Joe et al., 2009). In addition, the dietary supplementation of Zn from Bioplex Zn resulted in a higher (*P* < 0.05) content of Zn in the pancreas compared to the pancreatic Zn content of chicks fed a ZnO supplemented diet. This finding indicated that the bioavailability of Bioplex Zn was higher in 21-day-old chicks compared to the bioavailability of ZnO. In contrast to our findings, the supplementation of a corn soybean meal diet with 0, 30, 60, and 90 mg/kg organic Zn with different chelation strengths in broiler diets did not result in any significant difference in pancreas Zn concentration (Huang et al., 2009). These authors suggested that the pancreatic Zn concentrations from broiler chicks fed a diet supplemented with a chelated Zn source lacked the sensitivity to detect the difference among the evaluated sources.

### Excreta Zn Content

Data analysis indicated there was a (*P* < 0.05) main effect of Zn imprinting, source, and level on the excreta Zn content. The excreta Zn content was higher (*P* < 0.05) from birds fed the basal diet supplemented with 40 mg/kg of Zn compared to the excreta Zn content from birds fed the basal diet supplemented with 8 mg/kg Zn. These results were in agreement with other studies which indicated that feeding high levels of dietary Zn contributed to large quantities of Zn in the excreta (Carlson et al., 2004; Zhao et al., 2010; Aksu et al., 2011; Yuan et al., 2011). Additionally, Nollet et al. (2008) reported that the mineral excretion from broilers fed 100 and 67% of organic minerals based on the NRC (1994) requirement was not significantly different from the mineral excretion content from birds fed at the 100% level of inorganic minerals. These observations led to the conclusion that the supplementation of higher levels of organic minerals did not lead to higher mineral retention despite their higher bioavailability.

Bao et al. (2007) reported that the highest level of organic trace minerals did not contribute to broiler growth, but resulted in a higher excretion of the examined trace minerals. Results from our study indicated that it is possible to supplement trace minerals from inorganic sources as well as organic sources in low levels without compromising performance, subsequently helping to reduce the rate of trace mineral excretion. The lower excreta Zn concentrations from the Zn imprinted broiler chicks reflect the ability of broiler chicks to adapt to a low Zn diet. This is associated with increased Zn absorption, and the reduction in the excreta Zn content to conserve and maintain normal Zn levels for vital functions in the body (Attia et al., 2013).

In summary, under the experimental conditions of this study, it can be concluded that early Zn imprinting (during 1 to 4 d) can affect body Zn stores and improve performance of broiler chicks. Analysis of tissue mineral content suggested that the bioavailability of Bioplex Zn and ZnO was the same although Bioplex Zn was numerically higher than ZnO. There was no clear evidence that performance was affected by replacing inorganic Zn with organic Zn. However, the use of 8 mg/kg of Bioplex Zn promoted improved feed efficiency when compared to the use of 40 mg/kg of Bioplex Zn but this improved efficiency was not significantly different from the use of 8 and 40 mg/kg of ZnO. The results observed in this study demonstrated that post hatch nutrients can have prolonged effect on broiler performance and tissue mineralization. However, further studies are required to determine the practical application of Zn imprinting in commercial industry.
